# Combination treatment with anti-HER2 therapeutic antibody RC48, PD-1 inhibitor, radiotherapy, and granulocyte macrophage-colony stimulating factor (GM-CSF) in patient with metastatic gastric cancer: a case report

**DOI:** 10.3389/fimmu.2024.1321946

**Published:** 2024-02-01

**Authors:** Zhuixing Liu, Fang Wang, Yingqi Zhang, Jun Lu, Yang Yang

**Affiliations:** ^1^ Department of Oncology, Xi‘an International Medical Center Hospital, Xi‘an, China; ^2^ Department of Radiotherapy & Oncology, Xi‘an International Medical Center Hospital, Xi‘an, China

**Keywords:** RC48, PD-1 inhibitor, stereotactic body radiotherapy, GM-CSF, gastric cancer, HER2-positive

## Abstract

HER2 overexpression/amplification is a prevalent driver in various types of cancer, including gastric cancer (GC). Limited options are available for patients with HER2-positive metastatic gastric cancer, particularly those who do not respond to the standard therapy of HER2 antibody trastuzumab combined with chemotherapy. Previous research suggests that combining a PD-1 inhibitor with radiotherapy and granulocyte macrophage-colony stimulating factor (PRaG regimen) may enhance the antitumor effects in patients with chemotherapy-resistant metastatic solid tumors. In this case study, we presented a potential treatment strategy of a patient having HER2-positive and PD-L1-negative gastric adenocarcinoma. The patient showed rapid tumor progression even after surgery and multiple trastuzumab plus chemotherapy treatments. To address this, we employed a novel anti-HER2 antibody called RC48 in combination with PRaG regimen therapy (PRaG3.0). The patient demonstrated a positive response after two treatment cycles and achieved a progression-free survival time of 6.5 months. This case highlights the potential of four-combination therapies for treating refractory, multiorgan, HER2-positive, PD-L1-negative metastatic gastric cancer. Additionally, varying radiation doses in targeting dual foci is critical to enhance tumor immunotherapy.

## 1 Introduction

According to the findings from Global Cancer Statistics 2020, gastric cancer (GC) is the fifth most commonly diagnosed cancer and the fourth leading cause of cancer-related deaths worldwide (1). In China, there are over 20,000 new cases of GC each year, with approximately 170,000 deaths attributed to this disease, accounting for about 25% of all cancer-related deaths (2). Around 20% of GC patients show an overexpression of human epidermal growth factor receptor 2(HER2) (3). The phase III ToGA trial has confirmed trastuzumab as the approved medication to be included in the initial chemotherapy regimen for advanced GC patients with HER2 overexpression (4). The Chinese population has further supported the effectiveness and safety of trastuzumab through a non-interventional registry study called EVIDENCE (5). Unfortunately, treatment options for patients with metastatic GC who do not respond to conventional chemotherapy are very limited, and the prognosis is poor (6). However, a promising treatment option called RC48, a novel anti-HER2 antibody, has shown encouraging results as a third-line or subsequent therapy for Chinese patients with HER2 overexpressing GC. The objective response rate was found to be 24.8% (95%CI:17.5%-33.3%), and the median progression-free survival (PFS) was 4.1 months (95%CI:3.7-4.9 months) (7).

In recent times, the rapid advancement of immune checkpoint inhibitors (ICIs) has revealed the potent effectiveness and tolerable side effects of immunotherapy in treating metastatic GC (8, 9). Specifically, Nivolumab and pembrolizumab have gained approval for managing advanced and recurring GC. Nevertheless, the effectiveness of immunotherapy as a monotherapy stands at a mere 15-25% (10). Particularly, when it comes to refractory metastatic gastric cancer that tests negative for programmed death ligand 1 (PD-L1), the efficacy of PD-1/PD-L1 inhibitors alone drops even further.

Radiation therapy has the potential to elicit immunogenic cell death, release various cytokines and chemokines, and induce an increase in PD-L1 expression (11, 12). Compared to traditional radiotherapy, stereotactic body radiotherapy (SBRT) that delivers more than 5Gy of radiation can enhance the immune response. Therefore, combining SBRT with immunotherapy might be more effective than the combination of conventional fractionated radiotherapy and immunotherapy (13).

Furthermore, it has been observed that when chemotherapy is combined with radiotherapy, it can lead to the disruption of MMR-related genes, resulting in changes in the MSI status (14). Based on these mechanisms, radiotherapy has the potential to convert cold tumors into hot tumors (15). The presence of granulocyte-macrophage colony-stimulating factor (GM-CSF) may facilitate the differentiation, maturation, recruitment, and activation of dendritic cells (DCs).As a result, the anti-tumor effects of radiotherapy and immunotherapy can be enhanced (16, 17). RC48, a recombinant humanized anti-HER2 antibody conjugated with monomethyl auristatin E, has demonstrated high efficacy in HER2-positive gastric cancer (7). A recent study by Hong Xu et al. reported a case of a patient with mediastinal lymph node metastasis who achieved complete remission (CR) following PRaG triple-combination therapy for postoperative gastric cancer (18). This case serves as evidence for the effectiveness of combining anti-PD-1 inhibitor immunotherapy with SBRT, GM-CSF, and RC48 therapy(PRaG3.0, NCT05115500) in HER2-positive refractory multiorgan metastatic gastric cancer patients.

## 2 Case presentation

**Figure 1:**
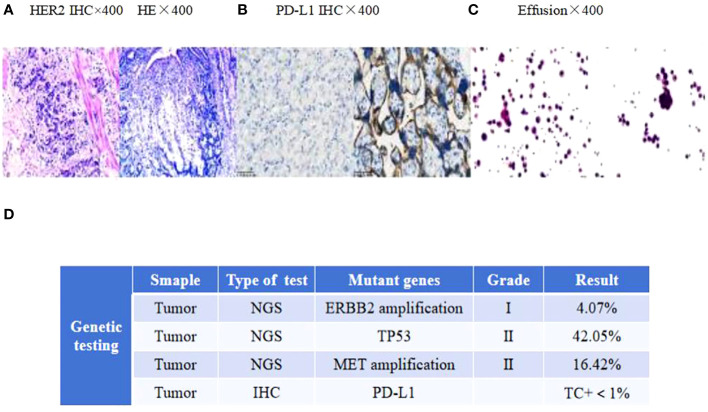
**(A)** HER2 and H&E staining of the primary tumor. **(B)** PD-L1 immunohistochemistry of mediastinal lymph nodes. **(C)** Adenocarcinoma tumor cells in the effusion. Scale bars: 25 µm. H&E: hematoxylin and eosin. **(D)** The next-generation sequencing (NGS) analysis of tumor DNA from the enlarged lymph nodes showed negative expression of PD-L1, gene mutations of TP53, gene amplification of ERBB2 and MET.

A 58-year-old man with upper abdominal pain was admitted to a local hospital on 11 November 2020.Gastroscopy revealed irregular protuberance and shallow ulcers at the gastric angle, resulting in a less curved gastric sinus. Pathology findings suggested a poorly differentiated gastric adenocarcinoma. The patient underwent a Billroth II subtotal gastrectomy at this hospital, and the subsequent pathological examination confirmed an ulcerative type of poorly differentiated gastric adenocarcinoma. Immunohistochemical analysis showed the following parameters: HER2:3+ and mismatch repair proficiency (pMMR) phenotype(MLH1/MSH2/MSH6/PMS2) (**Figure 1A**). Based on the surgical pathology information, the postoperative diagnosis was pT3N3aM0 (p-stage IIIB).

**Figure 2:**
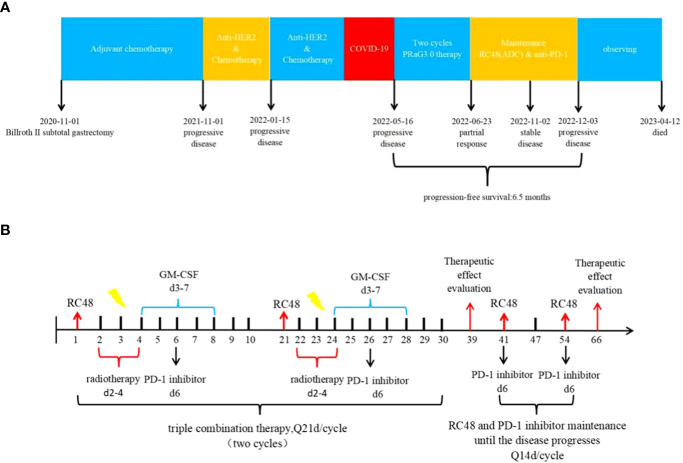
**(A)** Timeline of the whole treatment process for the patient. The patient experienced PD with the new emergence of mass in the left lobe of the liver during postoperative adjuvant chemotherapy on 01 November 2021.Two treatment cycles after trastuzumab with docetaxel plus cisplatin, the CT scan revealed metastases in both lungs, liver, thoracic cavity, hilar liver, head of pancreas, retroperitoneum, and parietal aorta on 15 January 2022.The treatment regimen was changed to trastuzumab plus irinotecan, but due to the COVID-19 pandemic, no further treatment was administered after only one cycle. On 16 May 2022, Baseline CT scans showed significant enlargement of tumor lesions in both lungs, mediastinal lymph nodes, liver metastases lesions. After 2 cycles of PRaG3.0 therapy, the sum of diameters of the unirradiated target metastases decreased by 58% from baseline. The patient was assessed as PR on 23 June 2022.The patient continue receiving RC48 and camrelizumab as maintenance treatment, with each cycle lasting for 2 weeks. Until 3 December 2023, when the patient suffered PD again and opted for supportive care. PD, progressive disease; PR, partial response; PFS, progression-free survival. **(B)** The patient underwent the first cycle of RC48 treatment (120mg on day 1) in combination with SBRT (24 Gy in 3 fractions for lung metastasis and 15 Gy in 3 fractions for partial liver metastasis on days 2-4). Additionally, GM-CSF (300ug on days 4-8) and camrelizumab (200mg on day 6) were administered. This treatment course was repeated every 3 weeks. Two courses of four-combination therapy were administered in total, targeting different metastases with SBRT. Subsequently, the patient received twelve cycles of RC48 (120mg on day 1) in combination with camrelizumab (200mg on day 1) after completing the four-combination therapy.

From 14 January 2021 to 20 June 2021, the patient underwent adjuvant chemotherapy with a regimen of S-1 (60 mg twice daily on days 1-14) plus oxaliplatin (100 mg on days 1-2) (SOX) for six cycles. However, a CT scan performed 11 months after surgery revealed nodules in the abdominal aorta and a mass in the left lobe of the liver. No medical treatment was administered, and one month later, a mass was detected in the upper lobe of the left lung, the left lobe of the liver, and multiple enlarged lymph nodes in the abdominal aorta. Consequently, first-line therapy was initiated on 13 November 2021,consisting of trastuzumab (600 mg on day 1) in combination with docetaxel (120 mg on day 1) plus cisplatin (120 mg on day 1). However, after two cycles, a follow-up CT scan revealed metastases in both lungs, liver, thoracic cavity, hilar liver, head of pancreas, retroperitoneum, and parietal aorta. The efficacy of the treatment was assessed as disease progression (PD) according to Response Evaluation Criteria in Solid Tumors (RECIST v1.1).On 15 January 2022, the treatment regimen was changed to trastuzumab plus irinotecan, but due to the COVID-19 pandemic, no further treatment was administered after only one cycle. A summary of the patient’s treatment history is presented in **Figure 2A**.

**Figure 3:**
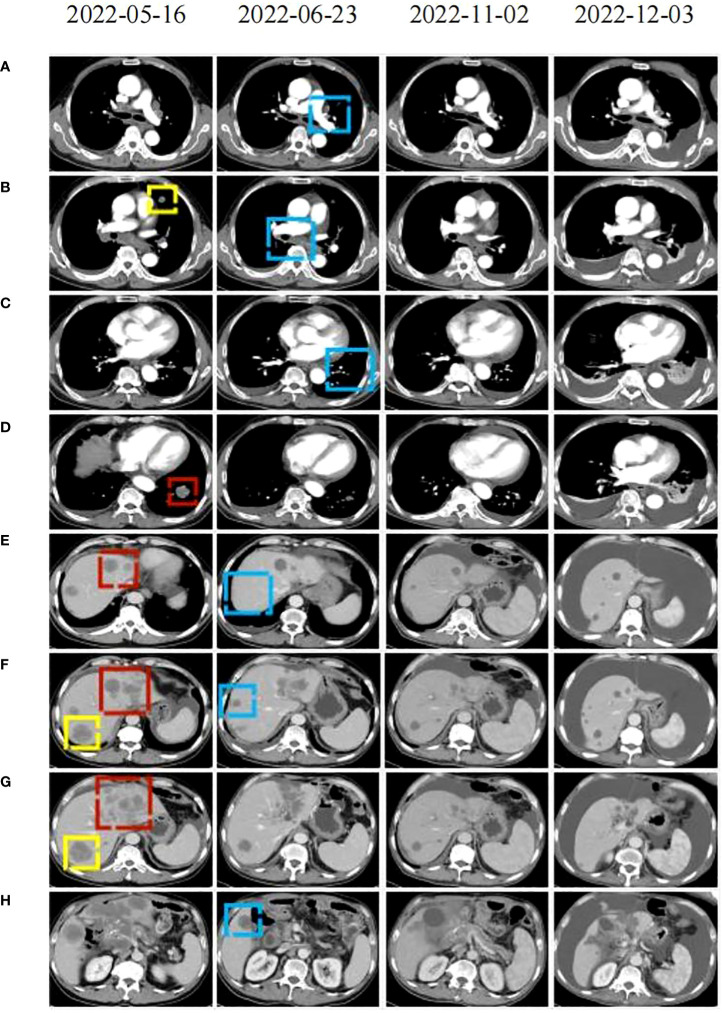
CT scans were conducted before (2022-05-16), during (2022-22-02), and after (2122-12-03, 2021-05-12) the PRaG3.0 therapy. The baseline inspection was performed on 2022-05-16 **(A–H)**: the red area indicates the first cycle of radiation, the yellow area represents the second cycle of radiation. The radiographic changes after two cycles of treatment were observed on 2022-06-23 **(A–H)**: the blue area indicating tumor regression in the unirradiated area. During maintenance treatment. On 2022-11-02 **(A–H)**: changes in the patient’s lesion were noted. On 2022-12-03 **(A–H)**, the tumor lesion progressed and thoracic and abdominal effusion developed.

**Figure 4:**
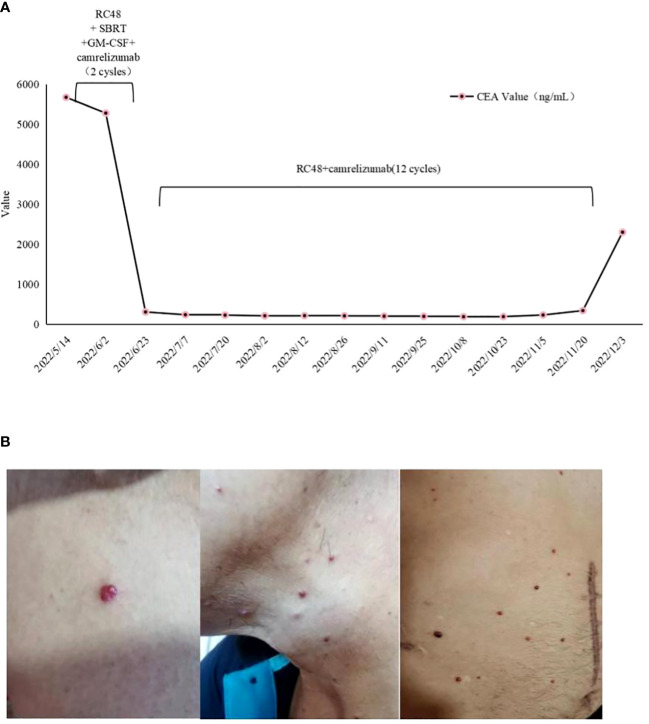
**(A)** Dynamics of cancer carcinoembryonic antigen (CEA) (ng/mL) levels during the entire disease course. **(B)** Management of irAE during the four-combination therapy. Representative images showing irAEs of **(B)** RCCEP, RCCEP, reactive cutaneous capillary endothelial proliferation.

In April 2022, the patient presented to our hospital with a left supraclavicular mass. Baseline CT scans showed significant enlargement of tumor lesions in both lungs, mediastinal lymph nodes, liver metastases lesions, and a multifold increase of carcinoembryonic antigen (CEA) to 5677 ng/mL. This was assessed as progressive disease (PD) and suggested that the tumor had high malignant behavior and was insensitive to trastuzumab chemotherapy agents (**Figure 3** 2022-05-16A-H, **Figure 4A**). The next-generation sequencing (NGS) analysis of tumor DNA from the enlarged lymph nodes in the mediastinal area 4 biopsy showed negative immunohistochemical level of PD-L1,gene mutations of TP53,gene amplification of HER2 and MET (**Figures 1B-D**). Finally, the patient was diagnosed with cTxN3M1 stage IV GC with an Eastern Cooperative Oncology Group (ECOG) performance status of 2. After consultation with the patient and their family, the PRaG3.0 combination treatment strategy was determined as the third-line therapy in order to expect a better survival benefit. On 16 May 2022, the patient underwent the first cycle of RC48 treatment (120mg on day 1). This treatment was combined with SBRT (24 Gy in 3 fractions) for the lesion in the inferior lobe of the left lung and (15 Gy in 3 fractions) for the partial left liver metastasis on days 2-4(**Supplementary Figures 1A, B**). Additionally, the patient received GM-CSF (300ug on days 4-8) and camrelizumab (200mg on day 6) based on family will, HER-2 and ECOG PS 2 score (**Figure 2B**). CT scans showed a 36% reduction in target lesions and the patient was assessed as partial remission (PR), with CEA levels decreasing to 1780 ng/mL (**Figure 4A**). After 21 days,the second cycle began, focusing on palliative radiotherapy for the metastasis in the upper lobe of the left lung (24 Gy in 3 fractions on days 3-5) and the partial lesion in the right liver (24 Gy in 3 fractions on days 3-5).Following two cycles, CT scans revealed regression of all metastases in multiple organs. The patient was again assessed as PR (**Figure 3** 2022-06-23A-H). However, the patient experienced grade 1 reactive cutaneous capillary endothelial proliferation (RCCEP), a novel immune-related adverse event (irAE) observed in the majority of patients treated with camrelizumab (19, 20) (**Figure 4B**). Based on the effectiveness of the regimen and the fact that the patient was in stage IV, we recommended that the patient continue receiving RC48 (120 mg on day 1) and camrelizumab (200 mg on day 1) as maintenance treatment, with each cycle lasting for 2 weeks. However, the patient experienced Grade 1 reactive RCCEP, leukopenia, anaemia, and thrombocytopenia during the treatment. On December 3, 2022, CT scans revealed increased liver metastases, a significant rise in CEA levels to 2303ng/mL, moderate hydrothorax, and severe hydroperitonium. Consequently, the patient was assessed as having progressive disease once again. To investigate further, an ultrasound puncture of the hydroperitonium was performed, which revealed the presence of adenocarcinoma tumor cells in the effusion (**Figure 1C**). Due to the lack of effective treatment options and the substantial economic burden, the patient refused subsequent antitumor treatment and opted for supportive care at a local hospital and unfortunately passed away in April 2023.

## 3 Discussion

Currently, China has a high number of gastric cancer cases and deaths, resulting in a heavy disease burden. Unfortunately, the 5-year survival rate for patients is just 35.9% (21). Most patients are diagnosed in the middle to late stages of the disease, leading to a preference for systemic therapy. Back in 2010,the ToGA study revealed that trastuzumab combined with chemotherapy could extend the survival of gastric cancer patients, marking it as the first-line treatment for HER2-positive patients (4). However, over the past decade, the second-line and subsequent treatments for HER2-positive gastric cancer have encountered obstacles, resulting in limited survival benefits for patients. Consequently, it is crucial to delve into new combination treatment strategies for better outcomes.

Drugs that specifically target HER2 comprise pertuzumab (JACOB study), lapatinib (TyTAN study and LOGIC study) (22, 23). Verdisitumab (C008 study) (7) and T-DXd (DESTINY-Gastric01 study) (24). Unfortunately, the results of the phase III study evaluating T-DM1 (a conjugate of trastuzumab and emtansine) as a second-line treatment for gastric cancer were unfavorable (25). The phase II C008 study, RC48 approval by the National Drug Administration came in June 2021. However, none of these medications presented a PFS exceeding 5 months. Consequently, the efficacy of single-agent anti-HER2 therapy remains limited.

From recent phase III trials like ATTRACTION-4 and CHECKMATE-649, it has been observed that the combination of first-line immunotherapy and chemotherapy offers survival benefits to individuals with metastatic gastric cancer. Moreover, this approach also presents a notable advantage in terms of the objective response rate (ORR) (26, 27). However, when it comes to the treatment of metastatic gastric cancer in later stages, specifically the third-line treatment, it becomes extremely challenging. Currently, the options available are nivolumab for HER2 negativity and RC48 for HER2 positivity. Unfortunately, none of these treatments have shown median survival rates exceeding 7 months (28, 29). The phase III KEYNOTE-062 study demonstrated that in patients with gastric cancer/gastric-esophageal junction with PD-L1 combined positive score(CPS)≥1,the median overall survival(OS) was 10.6 months for pembrolizumab alone and 11.1 months for chemotherapy alone. In the subgroup of gastric cancer patients with dMMR/MSI-H, a total of 14, 17, and 19 patients received pembrolizumab alone, pembrolizumab combined with chemotherapy, and chemotherapy alone, respectively. The ORR and 24-month survival rates were 57.1%/71%, 36.8%/26%, and 64.7%/65% respectively (30). These findings further support the superiority of immunotherapy alone or in combination with chemotherapy over chemotherapy alone, with a more pronounced long-term survival benefit observed with immunotherapy. As a PD-1 inhibitor developed in China, camrelizumab exhibits a significantly higher ORR rate when compared to chemotherapy alone (59.18% vs 38.89%), particularly when combined with chemotherapy (20). Additionally, the median PFS time for camrelizumab is 10.03 months compared to 6.24 months for chemotherapy alone. Subgroup analysis reveals that patients with PD-L1 CPS>1 experience an extended PFS, whereas those with PD-L1 CPS <1 have a poorer prognosis. The ORR rate of the two PD-1 inhibitors in gastric cancer patients was found to be comparable. Moreover, there was no significant difference in the efficacy of PD-1 inhibitors, including pembrolizumab, sintilimab, camrelizumab, toripalimab and tislelizumab, as observed in the PRaG regimen (31). Based on clinical practice in China and taking into account of patient’s financial burden, camrelizumab is considered one of the preferred drugs for third-line treatment in our patient. However, there is a need for further optimization and exploration of immunodrug treatment strategies in HER2-positive, PD-L1-negative advanced gastric cancer patients.

Tumor immunotherapy’s theoretical foundation elucidates the cytological mechanism by which the immune system eradicates tumors. Moreover, it offers novel research avenues for combined approaches in tumor immunotherapy. The tumor-specific immune response encompasses seven crucial processes, spanning from the tumor cells’ release of antigens to the clearance of tumor cells through T cell recognition (32). These consecutive steps form a recurrent cycle of tumor-immunity, where in each phase harmoniously regulates and maintains the overall circulation of tumor immunity. Therefore, the sole function of anti-PD-1/PD-L1 inhibitors is to alleviate the suppression of the immune system, whereas the most crucial aspect lies in directly eradicating cancerous cells through recognition of their T cell receptor, also known as T cell receptor (TCR), and a specific antigen that targets the tumor. Anti-PD-1/PD-L1 inhibitors are approved for treating patients exhibiting high levels of PD-L1 expression, high microsatellite instability (MSI-H)/mismatch repair deficiency (dMMR), or a high tumor mutation burden (TMB-H), irrespective of tumor type (33–35). However, in individuals with low PD-L1 expression, the ORR for using these inhibitors in monotherapy stands at a mere 15%-25% (4). Incorporating radiotherapy into the treatment regimen can escalate the efficacy to roughly 40% (36). Regardless, a significant proportion of cancer patients still fail to benefit from this combined therapeutic approach. Several factors contribute to this, including heterogeneity within tumor tissues, substantial tumor burden, the cold tumor microenvironment, as well as primary and secondary resistance to anti-PD-1/PD-L1 inhibitors, which collectively contribute to the low response rates observed (37, 38). Radiotherapy, a local therapy used to treat tumors, has the ability to enhance the effectiveness of immunotherapy. The abnormal expression of antigens on the surface of tumors, such as a decrease or alterations in major histocompatibility complex (MHC)-presenting antigens, leads to a decrease in T-cell activation and a deficiency of CD4+T cells and CD8+T cells within the tumor’s stromal tissue. Consequently, this allows tumor cells to evade the immune system’s response (39). One way that radiotherapy achieves this is by exposing tumor antigens and increasing MHC expression, thereby enhancing antigen presentation (40). In the PEMBRO-RT trial, the ORR was 36% for patients receiving pembrolizumab in combination with radiotherapy, while it was 18% for those receiving pembrolizumab alone. Although not statistically significant (P=0.07), this suggests that radiotherapy may improve the effectiveness of pembrolizumab in treating metastatic non-small-cell lung cancer. Additionally, patients who received the combination therapy had a mPFS of 6.6 months and a mOS of 15.9 months, compared to only 1.9 months and 7.6 months, respectively, for patients who received pembrolizumab alone. It’s worth noting that no additional side effects were observed with the combination therapy (41). Furthermore, a 5-year follow-up study evaluating the combination of radiotherapy and immunotherapy for metastatic solid tumors supported these findings, with a median PFS of 6.52 months and a median OS of 15.32 months (42).

GM-CSF has the ability to enhance the production of granulocytes, macrophages, and dendritic cells, thereby functioning as an immune adjuvant (43). In preclinical investigations using the B16 melanoma model, irradiated tumor cells alone failed to elicit significant anti-tumor immunity. However, irradiated tumor cells expressing GM-CSF stimulated robust and enduring anti-tumor immunity (16). A prospective clinical trial verifies that metastatic solid tumors treated with both radiotherapy and GM-CSF tend to experience tumor shrinkage (17). The increase in GM-CSF levels during radiotherapy correlates with longer PFS and OS in lung and esophageal cancer patients, indicating its potential as a prognostic biomarker (44). As distinct stages in triggering the immune response, radiotherapy, immunotherapy, and GM-CSF have the potential to enhance the efficacy against cancerous growths to different extents. In a study involving 54 solid tumors with multiple metastases that did not respond to standard treatment, patients were administered PD-1 inhibitors, radiotherapy and GM-CSF(PRaG1.0 regimen). The findings revealed that the objective response rate was 15.8% and the disease control rate was 46.3%. The median progression-free survival was 4.0 months (95% CI:3.3 to 4.8), and the median overall survival was 10.5 months (95% CI:8.7 to 12.2). These results demonstrated manageable toxicity and satisfactory short-term effectiveness (31). Additionally, in clinical studies, the inclusion of ADC and IL 2(PRaG3.0 regimen,NCT05115500) also exhibited favorable safety and efficacy. The results were also reported verbally on both ASCO and ASTRO. Consequently, the PRaG 3.0 regimen presents a novel therapeutic choice for the treatment of advanced HER2-positive patients.

ADC drugs have the ability to target the tumor area and deliver cytotoxic drugs. They can also target the HER2 protein on the tumor’s surface, accurately identify tumor cells, and expose other metastatic lesions to antigens (45). Radiotherapy plays a crucial role in optimizing the response of antigens and *in situ* vaccines by primarily reducing tumor burden and facilitating field binding (40, 41). It is anticipated that different doses of SBRT will further enhance this mechanism of action. This optimization can enhance the effectiveness of PD-1/PD-L1 inhibitors in combination with ADC. GM-CSF, a cytokine that regulates dendritic cell differentiation, proliferation, and survival, plays a crucial role in the *in situ* vaccine effect of radiation therapy and enhances the efficacy of immune checkpoint inhibitors (17, 43). This mechanism of action was considered in the research protocol.

Our study reveals that the patient exhibits a significant tumor burden, with a rapid decrease in tumor PR and CEA value after 2 treatment cycles (**Figure 3** 2022-06-23A-H and **Figure 4A**). Subsequently, during the late RC48 combined camrelizumab maintenance therapy, the tumor lesion remains in a stable condition (**Figure 3** 2022-11-02A-H), and the CEA value demonstrates a tendency towards normalization (**Figure 4A**). Importantly, we observe that irradiation at varying doses of dual foci plays a pivotal role in effectively controlling tumor regression (**Supplementary Figures 1A–D**). This finding urges us to further explore the optimal radiotherapy segmentation mode. The dose and fractionation schedule of radiotherapy in combination with immune checkpoint inhibitor have not yet been established and standardized. Preclinical studies have indicated that a hypofractionation regimen of 3 fractions of 5Gy each promotes antigen-presenting cell activation more effectively compared to a conventional fractionation of 5 fractions of 2Gy each (46). When a single radiotherapy fraction exceeds 5Gy, it demonstrates characteristics of an *in-situ* vaccine (47). Activation of adaptive immunity is observed with a moderate hypofractionation schedule of 2-3 fractions of 7.5-10Gy each, but higher fractional doses of 15Gy in a single fraction tend to compromise the promotion of the immune microenvironment (40). Clinical outcomes demonstrate favorable efficacy with the ultra-hypofractioned regimen of 3 fractions of 8Gy each combined with PD-1 inhibitors in PEMBRO-RT. Lung metastasis responds well to a fractionated regimen of 6 fractions of 10Gy each, whereas liver metastasis shows clinical viability with a fractionated regimen of 4 fractions of 12.5Gy each (41). Therefore, the segmentation and dose regimen used in this case (8 Gy×3f for lung metastasis, 5 Gy×3f for liver metastasis) may also be considered as an appropriate strategy for combined therapy.

When dealing with large masses or multiple instances of metastasis, it can be challenging to maintain safety due to the potential for dose-limiting toxicity. Also, radiation-induced depletion of lymphocytes may compromise the immune response (48). To address these concerns, it is more reasonable to irradiate only a portion of a large mass during each treatment cycle. Additionally, considering the heterogeneity of metastatic lesions, it is recommended to irradiate two or more metastases in different organs per cycle for multiple instances of metastasis. This approach ensures that all metastases are adequately controlled within a timely manner. As a result, several cycles of radiotherapy should be administered. It is important to note that irradiating lung metastases in combination with radiotherapy and immunotherapy has shown to prolong patient’s PFS (6.87 months vs. 5.63 months) and OS (18.67 months vs. 13.63 months), compared to irradiation of liver metastases (42). To our knowledge, this is the first documented case of using the PRaG 3.0 regimen, which involves simultaneous irradiation of two sites per cycle, to treat advanced gastric cancer. In our case, achieving a PFS of 6.5 months was possible by irradiating one lung metastasis and one liver metastasis per cycle.

In addition to a decrease in the size of the irradiated lesion, there is also a reduction of the unirradiated lesion, known as an abscopal effect (17). In our situation, the abscopal effect might involve immune sensitization induced by radiotherapy, although it cannot be ruled out that it was an immune enhancement promoted by RC48 and GM-CSF. A clinical trial that enrolled patients with advanced non-small cell lung cancer showed that PRaG 1.0 has a manageable safety profile, with fatigue, fever, and bone pain being the most prevalent side effects (49). Fatigue, anorexia, fever, and thyroid dysfunction were the most frequently observed treatment-related adverse events in phase II studies of metastatic solid tumors (31). The major adverse events associated with camrelizumab are RCCEP and hematological toxicity. RCCEP is the most common irAE observed in camrelizumab treatment, primarily affecting the skin with pathological manifestations of dermal capillary endothelial hyperplasia and capillary hyperplasia. The mechanism behind RCCEP is still unknown. According to the expert consensus of the Expert Committee of Anti-tumor Drug Safety Management of the Chinese Society of Clinical Oncology(CSCO),it is believed that RCCEP of grade 1-2 is usually self-limited, reversible, and predictable. Therefore, it is recommended to continue with ICIs (50). Several studies have shown a correlation between the development of RCCEP and the efficacy of camrelizumab. Patients with RCCEP tend to have better outcomes compared to those without RCCEP. For instance, in phase II studies of camrelizumab for advanced liver cancer, patients with RCCEP had a higher OS rate (19.3% vs 5.6%),longer PFS (3.2 months vs 1.9 months, HR=0.53),and better OS(17.0 months vs 5.8 months, HR=0.33) (51). In the phase III ESCORT study, the mOS for patients with RCCEP was 10.1 months compared to 2.5 months for patients without RCCEP (HR=0.21). This highlights the importance of continuing camrelizumab treatment after quadruple therapy (52).

However, the study protocol for this case differs slightly from that of PRaG 3.0.One major difference is that the cytokine IL-2 was not used, because our pharmacy did not purchase IL2.Despite this, we observed a significant reduction in tumor lesions after two cycles of treatment (**Figure 3** 2022-06-23A-H and **Figure 4A**). This deviation in our treatment protocol also distinguishes it from the PRaG 3.0 study protocol. The effectiveness of the PRaG study protocol is closely linked to the number of T lymphocytes, and IL-2 is crucial for enhancing the value, differentiation, and survival of T cells (53). One reason for the slightly inadequate treatment effect of our study protocol is the dose issue of GM-CSF. The PRaG 1.0 protocol recommended a dosage of 200ug of GM-CSF for 2 weeks (31), while the PRaG 3.0 protocol recommended a dosage of 200ug for 1 week and the study by Golden et al (17) was the use of GM-CSF 125ug/m^2^ for 2 weeks after the completion of radiotherapy. Referring to the recommended method of GM-CSF and the dose specification in our pharmacy, we chose to use GM-CSF 300ug on the third day after the start of radiotherapy for a duration of 1 week. This approach aims to reduce the inhibitory effect of radiotherapy on bone marrow. Additionally, different doses, sites, and methods of irradiation were considered. The PRaG study protocol typically uses a single-lesion multi-cycle irradiation mode with doses of 8Gy, 5Gy or 3Gy. However, in our study, we utilized a multi-cycle radiotherapy mode with different combinations of radiation doses for two lesions. Previous studies have shown that this approach can potentially enhance the effectiveness of PRaG Regimen, leading to a quantitative change in the abscopal effect (54). Currently, there are numerous ongoing clinical studies both domestically and internationally investigating the combination of SBRT irradiation with PD-1 inhibitors (NCT04535024, NCT02608385, NCT03275597, NCT02523313, NCT03391869).

## 4 Conclusion

The combination of PD-1 inhibitor with GM-CSF and RC48 therapy, along with SBRT, could potentially be used as treatment strategies for refractory multiorgan metastatic gastric cancer (GC) detected immunohistochemically with HER2 positivity and PD-L1 negativity. To determine the feasibility of these strategies, large-scale and prospective clinical trials will be necessary in the future.

## Data availability statement

The original contributions presented in the study are included in the article/**Supplementary Material**. Further inquiries can be directed to the corresponding author.

## Ethics statement

The studies involving humans were approved by Hospital Ethics Committee of Xi ‘an International Medical Center Hospital. The studies were conducted in accordance with the local legislation and institutional requirements. The participants provided their written informed consent to participate in this study. Written informed consent was obtained from the individual(s) for the publication of any potentially identifiable images or data included in this article.

## Author contributions

ZL: Writing – original draft. FW: Writing – original draft. YZ: Investigation, Project administration, Writing – original draft. JL: Writing – review & editing, Supervision. YY: Formal analysis, Investigation, Methodology, Validation, Writing – review & editing.
