# Supplementary material for: Combination treatment with anti-HER2 therapeutic antibody RC48, PD-1 inhibitor, radiotherapy, and granulocyte macrophage-colony stimulating factor (GM-CSF) in patient with metastatic gastric cancer: a case report
Source: Front Immunol. 2024 Feb 1;15:1321946. doi: 10.3389/fimmu.2024.1321946 (PMC10867122; doi:10.3389/fimmu.2024.1321946)
Supplement: Supplementary file 1 [file DataSheet_1.doc]

A B


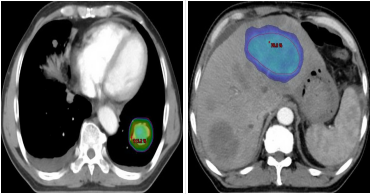


C D


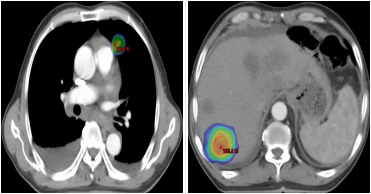


**Supplementary Figure 1.**In the first cycle of radiotherapy, (A) GTV: left lower lobe metastasis, PGTV: GTV extension 0.5cm; radiotherapy prescription dose: 24Gy/3f; (B) GTVm: partial metastasis of the liver left lobe; radiotherapy prescription dose: 15Gy/3f. In the second cycle of radiotherapy, (C)GTV: left upper lobe metastasis, PGTV: GTV extension 0.5cm; radiotherapy prescription dose: 24Gy/3f;(D)GTVm: right liver metastasis, PGTV extension 0.5cm, radiotherapy prescription dose: 15Gy/3f.

**Organs At Risk limits**

| **Three Fractions** |  |  |  | **Timmerman** |
| --- | --- | --- | --- | --- |
| **Serial Tissue** | **Volume** | **Volume Max(Gy)** | **Max Piont Dose**  **(Gy)**** | **Endpoint(≥Grade 3)** |
| Esophagus* | ＜5cc | 17.7Gy(5.9Gy/fx) | 25.2Gy(8.4Gy/fx) | Stenosis/fistula |
| Heart/Pericardium | ＜15cc | 24Gy(8Gy/fx) | 30Gy(10Gy/fx) | pericardtis |
| Great vessels | ＜10cc | 39Gy(13Gy/fx) | 45Gy(15Gy/fx) | aneurysm |
| Tracher and Large Bronchus* | ＜4cc | 15Gy(5Gy/fx) | 30Gy(10Gy/fx) | Stenosis/fistula |
| Bronchus-smaller airways | ＜0.5cc | 18.9Gy(6.3Gy/fx) | 23.1Gy(7.7Gy/fx) | Stenosis with atelectasis |
| Rib | ＜1cc | 28.8Gy(9.6Gy/fx) | 36.9Gy(12.3Gy/fx) | Pain of fracture |
| Skin | ＜10cc | 30Gy(10Gy/fx) | 33Gy(11Gy/fx) | ulceration |
| Stomach | ＜10cc | 16.5Gy(5.5Gy/fx) | 22.2Gy(7.4Gy/fx) | Ulceration/fistula |
| Bile duct |  |  | 35.7Gy(11.9Gy/fx) | stenosis |
| Duodenum* | ＜5cc  ＜10cc | 16.5Gy(5.5Gy/fx)  11.4Gy(3.8Gy/fx) | 22.2Gy(7.4Gy/fx) | ulceration |
| Jejunmum/Tlem* | ＜5cc | 24Gy(8Gy/fx) | 25.2Gy(8.4Gy/fx) | Enteritis/obstruction |
| Colon* | ＜20cc | 24Gy(8Gy/fx) | 28.2Gy(9.4Gy/fx) | Colitis/fistula |
|  |  |  |  |  |
| **Parallel Tissue** | **Critical**  **Volume(cc)** | **Critical Volume Dose Max(Gy)** |  | **Endpoint(≥Grade 3)** |
| Lung(Right & Left) | 1500cc | 10.5Gy(3.5Gy/fx) |  | Basic Lung Function |
| Lung(Right & Left) | 1000cc | 11.4Gy(3.8Gy/fx) |  | Pneumonitis |
| Liver | 700cc | 17.1Gy(5.7Gy/fx) |  | Basic Liver Function |
| Renal cortex(Right & Left) | 200cc | 14.4Gy(4.8Gy/fx) |  | Basic renal Function |
